# Enhancing deep learning in AI-enhanced education: a dual mediation model of cognitive load and learning motivation through interaction quality

**DOI:** 10.3389/fpsyg.2026.1768822

**Published:** 2026-03-05

**Authors:** Li Dong

**Affiliations:** Ningbo Childhood Education College, Ningbo, China

**Keywords:** cognitive load theory, covariance-based structural equation modeling, deep learning strategies, intelligent educational systems, interaction quality, learning motivation

## Abstract

This research develops and validates a dual mediation framework examining the pathways through which interaction quality in artificial intelligence educational systems is positively associated with deep learning outcomes via cognitive load reduction and motivational enhancement. Utilizing covariance-based structural equation modeling (CB-SEM), we analyzed survey data from 570 university teachers engaged with AI-powered learning platforms. Findings demonstrate that high-quality human-AI interaction significantly reduces cognitive burden, which in turn is positively related to learning motivation and shows pathways to deep learning approaches. Bootstrap procedures confirmed robust sequential mediation effects, with this pathway accounting for 53% of the total variance. The model achieved excellent fit indices and explained 31.5% of variance in deep learning outcomes. By synthesizing Cognitive Load Theory with Self-Determination Theory, this study contributes to educational technology scholarship by elucidating the psychological mechanisms linking interface design to learning depth. The empirical evidence provides actionable insights for developing AI educational systems that strategically minimize cognitive demands, foster motivational engagement, and support meaningful learning experiences.

## 1 Introduction

### 1.1 Research background and problem statement

The integration of artificial intelligence technologies into educational systems has accelerated dramatically across global higher education institutions, fundamentally transforming pedagogical practices and learning modalities (Homyamyen et al., 2025; Li and Jiang, 2025). Contemporary AI-enhanced learning environments encompass intelligent tutoring systems, adaptive learning platforms, and automated feedback mechanisms that create unprecedented opportunities for personalized instruction (Namaziandost, 2025; Hajian et al., 2025). However, this technological proliferation has generated critical questions regarding how teachers' interaction experiences with AI systems influence fundamental learning processes and outcomes.

Despite substantial investment in AI educational technologies, empirical understanding of the mechanisms through which human-AI interaction quality affects learning remains theoretically fragmented (Sun et al., 2025; Yuan et al., 2025). Existing research predominantly emphasizes technological capabilities and adoption behaviors, yet systematic investigation into the cognitive and motivational pathways connecting interaction experience to learning depth is conspicuously limited (Dong, 2025; Huang and Zhang, 2025). This theoretical gap is particularly problematic given that deep learning—characterized by critical thinking, knowledge integration, metacognitive awareness, and transfer application—represents essential competencies for contemporary knowledge economies (Grozev et al., 2024; Wang and McWatt, 2023).

Cognitive Load Theory (CLT) provides foundational insights into how instructional design influences learning effectiveness through management of limited working memory resources (Skulmowski and Xu, 2022; Kalyuga and Liu, 2015). In AI-mediated learning contexts, interface design and interaction modalities directly impact extraneous cognitive load, potentially either facilitating or hindering cognitive resource allocation toward meaningful learning (Schulz et al., 2025; Ngandoh et al., 2025). Concurrently, Self-Determination Theory (SDT) emphasizes that psychological need satisfaction—particularly autonomy and competence—fundamentally influences intrinsic motivation and sustained learning engagement (Akram and Li, 2024; Pan, 2023). However, these theoretical frameworks have rarely been integrated to examine AI educational systems, and the potential sequential relationships among interaction experience, cognitive processing, motivational states, and learning approaches remain empirically unexplored (Li et al., 2025; An et al., 2022).

Emerging evidence suggests that cognitive load may serve as a critical intermediary variable linking interface design quality to motivational processes, with excessive cognitive demands depleting psychological resources necessary for autonomous motivation (Fu et al., 2024; Tan et al., 2024). Furthermore, motivational states appear central to sustaining the deep cognitive processing characteristic of meaningful learning (Bailey et al., 2021; Khamparia and Pandey, 2020). Nevertheless, no empirical studies have systematically examined whether cognitive load and learning motivation function as sequential mediators in the pathway from human-AI interaction experience to deep learning outcomes. This theoretical integration gap limits both scholarly understanding of AI-enhanced learning mechanisms and practical guidance for educational technology design.

### 1.2 Research objectives and significance

This study constructs and empirically validates a sequential mediation model examining how human-AI interaction experience influences teachers' deep learning through cognitive load and learning motivation as consecutive intermediary mechanisms. Employing covariance-based structural equation modeling (CB-SEM) with data from higher education teachers engaged in AI-enhanced learning, the research systematically tests theoretically derived relationships among these constructs.

The theoretical contributions of this investigation are substantial. First, the study extends Cognitive Load Theory by demonstrating how human-AI interaction design constitutes a critical source of extraneous cognitive load in contemporary educational technologies, advancing understanding of cognitive load antecedents in AI-mediated contexts (Jose et al., 2025; Liu et al., 2025). Second, it deepens Self-Determination Theory by establishing cognitive resource availability as a prerequisite for motivational processes, revealing previously underexplored connections between cognitive and motivational theoretical frameworks (Chaidir et al., 2025; Syamala et al., 2025). Third, the research contributes to learning experience design scholarship by integrating user experience principles with cognitive and motivational theories, providing a unified framework for understanding technology-enhanced learning (Tawfik et al., 2024a; Salas et al., 2019).

From a practical perspective, empirical validation of the sequential mediation pathway offers evidence-based guidance for multiple stakeholder groups. Educational technology designers can leverage findings to optimize AI system interfaces that minimize cognitive load while supporting motivational needs (Lee and Hsu, 2021; Qasrawi et al., 2021). Instructional practitioners gain insights into designing learning activities that align with cognitive and motivational principles (Purnama et al., 2021; Kio and Lau, 2017). Institutional policymakers obtain evidence for resource allocation decisions regarding AI educational infrastructure and faculty professional development. Ultimately, the research illuminates systematic strategies for fostering deep learning approaches through coordinated attention to interaction experience quality, cognitive load management, and motivational support mechanisms.

This investigation addresses three fundamental research questions:

**RQ1**: How does human-AI interaction experience influence teachers' deep learning in AI-enhanced educational systems?

**RQ2**: What roles do cognitive load and learning motivation play in the relationship between human-AI interaction experience and deep learning?

**RQ3**: Do cognitive load and learning motivation constitute a sequential mediation chain linking human-AI interaction experience to deep learning?

## 2 Theoretical framework

### 2.1 Human-AI interaction experience in educational systems

Human-AI interaction experience encompasses learners' subjective evaluations of usability, interaction quality, system responsiveness, and satisfaction when engaging with AI-enhanced learning environments (Tawfik et al., 2024a; Syamala et al., 2025). Distinguished from conventional digital platforms, AI educational systems exhibit adaptive algorithms, intelligent feedback mechanisms, and personalized recommendation functions that fundamentally alter interaction dynamics (Namaziandost, 2025; Hajian et al., 2025). Interface design characteristics—including visual clarity, navigational coherence, and operational consistency—directly influence cognitive processing efficiency and user satisfaction (Salas et al., 2019; Qasrawi et al., 2021).

Empirical evidence indicates that superior interaction experience correlates positively with learning engagement and technology acceptance, while poorly designed interfaces generate cognitive frustration and disengagement (Yuan et al., 2025; Dong, 2025). However, the psychological mechanisms linking interaction quality to learning outcomes remain theoretically underspecified, necessitating systematic examination of cognitive and motivational pathways (Chaidir et al., 2025; Nguar and Appolloni, 2024).

### 2.2 Cognitive load theory and its application in AI-enhanced learning

Cognitive Load Theory posits that instructional effectiveness depends upon optimizing working memory capacity allocation across three load types: intrinsic load (inherent task complexity), extraneous load (imposed by instructional design), and germane load (facilitating schema construction) (Skulmowski and Xu, 2022; Kalyuga and Liu, 2015). In AI-mediated learning, interaction design directly influences extraneous load through interface complexity, information presentation formats, and navigational demands (Schulz et al., 2025; Ngandoh et al., 2025).

Well-designed human-AI interfaces minimize extraneous load by reducing unnecessary cognitive processing, thereby preserving working memory resources for meaningful learning activities (Meng et al., 2016; Lin et al., 2013). Conversely, algorithmic opacity, inconsistent feedback, and interface complexity impose additional cognitive burdens that may overwhelm learners' processing capacity (Jose et al., 2025; Liu et al., 2025). Critically, elevated cognitive load depletes psychological resources necessary for sustained motivation and deep cognitive engagement (Lee and Hsu, 2021; Khamparia and Pandey, 2020).

### 2.3 Learning motivation in technology-enhanced learning environments

Self-Determination Theory provides a robust framework for understanding motivational processes in educational contexts, emphasizing three fundamental psychological needs: autonomy (self-directed action), competence (perceived effectiveness), and relatedness (social connection) (Akram and Li, 2024; Pan, 2023). Intrinsic motivation—engagement driven by inherent interest and enjoyment—emerges when these needs are satisfied, promoting sustained learning investment and deep cognitive processing (An et al., 2022; Bailey et al., 2021).

In AI learning environments, system design characteristics influence need satisfaction through multiple pathways. Adaptive personalization may enhance autonomy by providing learner control, while intelligent feedback can strengthen competence perceptions through performance validation (Tan et al., 2024; Fu et al., 2024). However, cognitive load constitutes a critical antecedent condition: excessive cognitive demands deplete psychological resources, undermining perceived competence and autonomous motivation (Khamparia and Pandey, 2018; Kim and Lee, 2019).

### 2.4 Deep learning as educational outcome

Deep learning approaches encompass critical thinking, knowledge integration, metacognitive awareness, and transfer application, distinguished from surface learning characterized by memorization and reproduction (Grozev et al., 2024; Wang and McWatt, 2023). Empirical investigations demonstrate that deep learning correlates with superior knowledge retention, problem-solving capability, and adaptive expertise in novel contexts (Mphahlele, 2022; Shaari et al., 2012).

Motivational states fundamentally influence learning approach adoption (Jin et al., 2025; Santana et al., 2022; Tawfik et al., 2024b). Intrinsically motivated learners demonstrate greater propensity for deep processing, investing cognitive effort in understanding principles rather than superficial task completion (Homyamyen et al., 2025). Technology-mediated environments can facilitate deep learning through well-designed scaffolding and authentic tasks, yet effectiveness depends critically upon managing cognitive load and supporting motivational needs (Li et al., 2025; Ni et al., 2024).

### 2.5 Theoretical integration and hypothesis development

Integrating Cognitive Load Theory, Self-Determination Theory, and user experience research, this study proposes a sequential mediation model wherein human-AI interaction experience influences deep learning through consecutive effects on cognitive load and learning motivation.

**H1: Human-AI interaction experience negatively affects cognitive load**.

Well-designed AI interfaces characterized by usability, clarity, and responsiveness reduce extraneous cognitive load by minimizing unnecessary processing demands. Superior interaction quality streamlines cognitive resource allocation, enabling learners to focus on content comprehension rather than interface navigation.

**H2: Cognitive load negatively affects learning motivation**.

Elevated cognitive load depletes working memory and attentional resources, diminishing learners' perceived competence and autonomous engagement. When cognitive demands overwhelm capacity, psychological resources necessary for intrinsic motivation become unavailable.

**H3: Learning motivation positively affects deep learning**.

Intrinsic motivation promotes deep cognitive processing by sustaining attention, effort investment, and persistence in challenging learning tasks. Motivated learners actively seek meaning, integrate knowledge structures, and engage metacognitive monitoring—hallmarks of deep learning approaches.

The sequential mediation pathway posits that human-AI interaction experience influences deep learning through a theoretically integrated chain: interaction experience reduces cognitive load, thereby preserving psychological resources for autonomous motivation, which subsequently facilitates deep learning engagement. This framework reconciles technological, cognitive, and motivational perspectives within a unified explanatory model.

However, the integration of AI tools in higher education introduces specific theoretical tensions not fully captured by traditional CLT-SDT frameworks. First, AI-generated content raises concerns about accuracy and reliability, which may paradoxically increase cognitive load through verification demands while simultaneously reducing task complexity. Second, the autonomy dimension of SDT faces new challenges: while AI tools may enhance perceived control over pedagogical tasks, they may also create dependency that undermines professional autonomy. Third, ethical considerations—including data privacy, algorithmic transparency, and the delegation of educational judgment to AI systems—represent boundary conditions that may moderate the proposed relationships. These AI-specific factors position the current study at the intersection of cognitive, motivational, and socio-technical concerns in educational technology adoption.

## 3 Method

### 3.1 Research design and participants

This study employed a cross-sectional survey design to examine relationships among human-AI interaction experience, cognitive load, learning motivation, and deep learning in AI-enhanced educational contexts (Grozev et al., 2024). The target population comprised higher education teachers who had utilized AI educational systems for at least one academic semester, ensuring sufficient exposure to develop stable perceptions of interaction experience.

Participants were recruited through stratified random sampling across multiple universities, with stratification criteria including institutional type, academic discipline, and enrollment level (Akram and Li, 2024). Sample size determination followed Hair et al. guidelines for covariance-based structural equation modeling, requiring a minimum 10:1 ratio of observations to estimated parameters, coupled with power analysis specifications (α = 0.05, power = 0.80) for detecting medium effect sizes (Yuan et al., 2025).

Inclusion criteria specified: (1) a current teaching position in higher education institutions, (2) minimum one semester of AI educational system usage, (3) age 18 years or above, and (4) provision of voluntary informed consent. Exclusion criteria eliminated incomplete questionnaires, responses exhibiting systematic patterns indicative of inattentive responding, and completion times falling outside predetermined acceptable ranges (Dong, 2025). Ethical approval was obtained from the institutional review board prior to data collection.

### 3.2 Measurement instruments

All constructs were operationalized using validated psychometric instruments adapted for AI educational contexts. Responses were recorded on seven-point Likert scales (1 = strongly disagree, 5 = strongly agree) to maximize measurement precision and variance (Namaziandost, 2025).

Human-AI Interaction Experience Scale. This construct was assessed using eight items adapted from the User Experience Questionnaire and Technology Acceptance Model, measuring perceived usability, interaction quality, system responsiveness, and overall satisfaction with AI learning systems (Kio and Lau, 2017). The scale captured learners' evaluations of interface clarity, navigational ease, feedback appropriateness, and general interaction satisfaction.

Cognitive Load Scale. Seven items derived from established cognitive load instruments assessed three load dimensions: intrinsic load (task complexity), extraneous load (interface-imposed demands), and germane load (schema construction) (Skulmowski and Xu, 2022). Items measured mental effort requirements, processing difficulty, and cognitive burden associated with AI system usage.

Learning Motivation Scale. Seven items adapted from the Academic Self-Regulation Questionnaire measured intrinsic motivation and autonomous regulation in AI-mediated learning (Pan, 2023). The scale assessed inherent enjoyment, interest-driven engagement, and self-directed learning behaviors within technology-enhanced environments.

Deep Learning Scale. Eight items derived from the Revised Study Process Questionnaire evaluated deep learning approaches, including critical thinking, knowledge integration, reflective analysis, and metacognitive awareness (Wang and McWatt, 2023). Items assessed learners' propensity to seek underlying meanings, relate concepts across domains, and engage in critical evaluation of AI-provided information.

All instruments underwent pilot testing with an independent sample to verify content validity, item clarity, and cultural appropriateness. Minor linguistic adjustments were implemented based on pilot feedback without altering construct operationalization (Homyamyen et al., 2025).

### 3.3 Data collection procedure

Data collection occurred from February 15, 2025 to May 31, 2025, during the spring semester, strategically avoiding examination periods to minimize stress-induced response (Li and Jiang, 2025). An online survey platform facilitated questionnaire administration, enabling automated quality controls and response monitoring.

Multiple quality assurance mechanisms were implemented. Attention check items identified inattentive respondents (Tan et al., 2024). Reverse-coded items within each scale detected acquiescence bias and systematic response patterns. Completion time parameters flagged potentially invalid responses falling below minimum threshold (5 min) or exceeding maximum duration (30 min). IP address verification prevented duplicate submissions, while CAPTCHA authentication eliminated automated responses (Purnama et al., 2021).

Recruitment emails containing study information, consent procedures, and survey access links were distributed to eligible participants. Reminder communications were sent at 7 day intervals to enhance response rates. Participants completing the questionnaire were entered into a random drawing for electronic gift cards as participation incentive (An et al., 2022).

### 3.4 Data analysis strategy

Data analysis proceeded through sequential stages employing SPSS 27.0 for preliminary analyses and AMOS 26.0 for structural equation modeling (Nguar and Appolloni, 2024).

Data Screening and Preparation. Missing data patterns were examined to determine randomness and extent. Cases with missing data were retained and handled through Full Information Maximum Likelihood (FIML) estimation to preserve sample size and minimize bias (Sun et al., 2025). Multivariate outliers were identified using Mahalanobis distance criterion (χ^2^, *p* < 0.001), with flagged cases removed following case-by-case examination. Univariate normality was assessed through skewness and kurtosis indices, with absolute values exceeding 2.0 and 7.0 respectively indicating violation of normality assumptions (Huang and Zhang, 2025).

Measurement Model Assessment. Confirmatory factor analysis evaluated the hypothesized four-factor measurement model. Internal consistency reliability was assessed using Cronbach's alpha coefficient (α > 0.70) and composite reliability (CR > 0.70) thresholds (Bailey et al., 2021). Convergent validity required average variance extracted (AVE) exceeding 0.50 for each construct. Discriminant validity was established through the Fornell-Larcker criterion, requiring the square root of AVE for each construct to exceed its correlations with other constructs. Model fit was evaluated using multiple indices: χ^2^/df < 3.0, comparative fit index (CFI > 0.90), Tucker-Lewis index (TLI > 0.90), root mean square error of approximation (RMSEA < 0.08), and standardized root mean square residual (SRMR < 0.08) (Chaidir et al., 2025).

Common Method Bias Assessment. Harman's single-factor test examined whether a single factor accounted for majority variance in all measured variables, with variance proportions below 50% indicating acceptable common method bias levels (Khamparia and Pandey, 2020). Additionally, common latent factor analysis assessed whether incorporating a latent method factor substantially altered structural path coefficients, with changes below.02 considered negligible.

Structural Model and Hypothesis Testing. The hypothesized structural model specified direct paths from human-AI interaction experience to cognitive load (H1), cognitive load to learning motivation (H2), and learning motivation to deep learning (H3). Maximum likelihood estimation generated standardized path coefficients (β), standard errors (SE), critical ratios (CR), and significance levels (*p*-values) (Akram and Li, 2024). Model fit indices identical to measurement model criteria evaluated structural model adequacy. Coefficients of determination (*R*^2^) quantified proportions of variance explained in endogenous variables.

Mediation Analysis. Sequential mediation effects were examined using bootstrap methodology with 5,000 resamples and bias-corrected 95% confidence intervals (Homyamyen et al., 2025). Three indirect effect pathways were estimated: (1) human-AI interaction experience → cognitive load → learning motivation, (2) cognitive load → learning motivation → deep learning, and (3) human-AI interaction experience → cognitive load → learning motivation → deep learning (sequential mediation). Indirect effects were considered statistically significant when 95% confidence intervals excluded zero. Variance accounted for (VAF) quantified the proportion of total effect transmitted through indirect pathways, with established thresholds: VAF > 0.80 indicating full mediation, 0.20 < VAF < 0.80 indicating partial mediation, and VAF < 0.20 indicating minimal mediation (Kio and Lau, 2017).

## 4 Limitations

Several limitations should be noted. This study did not include control variables such as prior AI experience, discipline, institutional context, or instructor demographics. This decision prioritized theoretical parsimony and focused on core psychological mechanisms derived from CLT and SDT. However, these contextual factors may moderate the observed relationships and warrant investigation in future research. Additionally, the 31.5% variance explained in deep learning suggests that other factors beyond the current model contribute substantially to learning outcomes.

## 5 Results

### 5.1 Preliminary analysis

The final analytical sample comprised 570 participants following data screening procedures. Descriptive statistics and bivariate correlations among study variables are presented in Table 1. Human-AI interaction experience demonstrated a mean of 3.82 (SD = 0.64), indicating moderate-to-positive perceptions of AI system interaction quality. Cognitive load exhibited a mean of 2.91 (SD = 0.88), suggesting relatively low cognitive burden among participants. Learning motivation (M = 3.56, SD = 0.71) and deep learning (M = 3.74, SD = 0.79) both reflected moderate-to-high levels. All variables demonstrated acceptable univariate normality, with skewness and kurtosis values falling within established thresholds.

**Table 1:** Descriptive statistics and correlation matrix.

**Variable**	***M***	**SD**	**1**	**2**	**3**	**4**
1. Human-AI Interaction Experience	3.82	0.64	-			
2. Cognitive Load	2.91	0.88	−0.428^***^	-		
3. Learning Motivation	3.56	0.71	0.258^***^	−0.316^***^	-	
4. Deep Learning	3.74	0.79	0.103^*^	−0.089^*^	0.499^***^	-

*N* = 570. *M*, mean; SD, standard deviation. ^*^p < 0.05. ^**^p < 0.01. ^***^p < 0.001 (two-tailed).

Correlation analysis revealed theoretically consistent patterns (Table 1). Human-AI interaction experience exhibited significant negative correlation with cognitive load (*r* = −0.428, *p* < 0.001), supporting the proposition that superior interaction design reduces cognitive burden. Cognitive load demonstrated significant negative correlations with learning motivation (*r* = −0.316, *p* < 0.001) and deep learning (*r* = −0.089, *p* < 0.05), indicating that elevated cognitive demands undermined motivational and learning processes. Learning motivation showed strong positive correlation with deep learning (*r* = 0.499, *p* < 0.001), consistent with theoretical predictions. Human-AI interaction experience correlated positively with learning motivation (*r* = 0.258, *p* < 0.001) and weakly with deep learning (*r* = 0.103, *p* < 0.05), suggesting potential indirect effect pathways.

### 5.2 Measurement model assessment

Confirmatory factor analysis evaluated the hypothesized four-factor measurement model comprising human-AI interaction experience, cognitive load, learning motivation, and deep learning. The measurement model demonstrated excellent fit to the data: χ^2^(402) = 419.67, *p* = 0.262; CFI = 0.998; TLI = 0.998; RMSEA = 0.009; SRMR = 0.028. All fit indices substantially exceeded conventional acceptability thresholds, indicating strong correspondence between theoretical structure and empirical data (Chaidir et al., 2025).

Factor loadings for all observed indicators exceeded the minimum threshold of 0.60, ranging from 0.632 to 0.787 for human-AI interaction experience, 0.740 to 0.844 for cognitive load, 0.731 to 0.796 for learning motivation, and 0.781 to 0.826 for deep learning (Table 2). All loadings achieved statistical significance (*p* < 0.001), confirming that observed indicators reliably reflected their respective latent constructs.

**Table 2:** Measurement model assessment.

**Construct**	**Items**	**Loading range**	**α**	**CR**	**AVE**	**√AVE**
Human-AI Interaction Experience	8	0.632-0.787	0.760	0.802	0.525	0.725
Cognitive Load	7	0.740-0.844	0.917	0.928	0.648	0.805
Learning Motivation	7	0.731-0.796	0.704	0.781	0.506	0.711
Deep Learning	8	0.781-0.826	0.935	0.941	0.672	0.820

*N* = 570. α = Cronbach's alpha; CR, composite reliability; AVE, average variance extracted; √AVE, square root of AVE. Model fit: χ^2^(402) = 419.67, *p* = 0.262; CFI = 0.998; TLI = 0.998; RMSEA = 0.009; SRMR = 0.028. All factor loadings significant at *p* < 0.001.

Reliability assessment demonstrated satisfactory internal consistency across all constructs (Table 2). Cronbach's alpha coefficients ranged from 0.704 to 0.935, with three of four constructs exceeding 0.75. Composite reliability values ranged from 0.781 to 0.941, all surpassing the 0.70 threshold. These results confirmed adequate measurement reliability (Bailey et al., 2021).

Convergent validity was established through average variance extracted (AVE) values ranging from 0.506 to 0.672, all exceeding the 0.50 criterion (Table 2). Discriminant validity was confirmed through the Fornell-Larcker criterion: the square root of AVE for each construct exceeded its correlations with all other constructs. Specifically, √AVE values were.725 (human-AI interaction experience), 0.805 (cognitive load), 0.711 (learning motivation), and 0.820 (deep learning), all exceeding the maximum inter-construct correlation of 0.499. These results confirmed that constructs captured distinct theoretical domains (Akram and Li, 2024).

### 5.3 Common method bias test

Harman's single-factor test indicated that the first unrotated factor accounted for 37.6% of total variance, substantially below the 50% threshold. This result suggested that common method bias did not constitute a major threat to validity (Khamparia and Pandey, 2020). Supplementary common latent factor analysis revealed that including a method factor produced path coefficient changes below 0.02, with standardized method factor loadings not exceeding0.30. These convergent results indicated that common method variance remained within acceptable limits and did not substantially compromise interpretation of structural relationships.

### 5.4 Structural model and hypothesis testing

The hypothesized structural model examining relationships among human-AI interaction experience, cognitive load, learning motivation, and deep learning demonstrated excellent fit: χ^2^(404) = 422.36, *p* = 0.282; CFI = 0.998; TLI = 0.998; RMSEA = 0.009; SRMR = 0.029 (Table 3). All fit indices met or exceeded recommended criteria, indicating strong model-data correspondence (Yuan et al., 2025).

**Table 3:** Structural model path coefficients and hypothesis testing.

**Hyp**.	**Path**	**β**	***SE***	***t***	***p***	**95% CI**	**Result**
H1	HAIE → CL	−0.488^***^	0.037	−13.19	< 0.001	[−0.557, −0.413]	Supported
H2	CL → LM	−0.357^***^	0.040	−8.93	< 0.001	[−0.436, −0.276]	Supported
H3	LM → DL	0.561^***^	0.033	17.00	< 0.001	[0.494, 0.624]	Supported

*N* = 570. HAIE, Human-AI Interaction Experience; CL, Cognitive Load; LM, Learning Motivation; DL, Deep Learning. Hyp, Hypothesis; β = standardized path coefficient; SE, standard error; CI = confidence interval. Model fit: χ^2^(404) = 422.36, *p* = 0.282; CFI = 0.998; TLI = 0.998; RMSEA = 0.009; SRMR = 0.029. R^2^: CL = 0.238; LM = 0.127; DL = 0.315. ^***^p < 0.001.

H1 posited that human-AI interaction experience negatively affects cognitive load. This hypothesis received strong empirical support [β = −0.488, SE = 0.037, *t* = −13.19, *p* < 0.001, 95% CI (-0.557, −0.413)]. The standardized path coefficient indicated that each standard deviation increase in interaction experience quality corresponded to approximately half a standard deviation decrease in cognitive load, explaining 23.8% of cognitive load variance (*R*^2^ = 0.238).

H2 predicted that cognitive load negatively affects learning motivation. Analysis confirmed this relationship [β = −0.357, SE = 0.040, *t* = −8.93, *p* < 0.001, 95% CI (−0.436, −0.276)]. The negative coefficient demonstrated that elevated cognitive load substantially diminished learning motivation. Cognitive load accounted for 12.7% of learning motivation variance (*R*^2^ = 0.127).

H3 hypothesized that learning motivation positively affects deep learning. This relationship received robust empirical validation [β = 0.561, SE = 0.033, *t* = 17.00, *p* < 0.001, 95% CI (0.494, 0.624)]. Learning motivation emerged as a strong predictor of deep learning approaches, explaining 31.5% of deep learning variance (R^2^ =0.315). This substantial effect magnitude underscored motivation's central role in promoting deep cognitive processing.

All three hypothesized direct paths achieved statistical significance with substantial effect sizes, providing comprehensive support for the theoretical model's structural relationships (Table 3).

### 5.5 Mediation analysis

Bootstrap analysis with 5,000 resamples examined three indirect effect pathways (Table 4). Indirect Effect 1 tested whether cognitive load mediated the relationship between human-AI interaction experience and learning motivation. Results confirmed significant mediation [estimate = 0.186, SE = 0.028, 95% CI (0.135, 0.248)]. The entirely positive confidence interval indicated that superior interaction experience enhanced motivation by reducing cognitive load.

**Table 4:** Mediation effects analysis (bootstrap method).

**Effect path**	**Estimate**	***SE***	**95% CI lower**	**95% CI upper**
Indirect Effect 1: HAIE → CL → LM	0.186^***^	0.028	0.135	0.248
Indirect Effect 2: CL → LM → DL	−0.211^***^	0.032	−0.280	−0.154
Sequential Mediation: HAIE → CL → LM → DL	0.118^***^	0.020	0.085	0.162

*N* = 570. Estimates based on 5,000 bootstrap resamples with bias-corrected confidence intervals. HAIE, Human-AI Interaction Experience; CL, Cognitive Load; LM, Learning Motivation; DL, Deep Learning; SE, standard error; CI, confidence interval. Effects are significant when 95% CI does not include zero. ^***^p < 0.001.

Indirect Effect 2 examined whether learning motivation mediated the relationship between cognitive load and deep learning. This pathway demonstrated significant mediation [estimate = −0.211, SE = 0.032, 95% CI (−0.280, −0.154)]. The negative coefficient reflected that cognitive load undermined deep learning through diminished motivation.

Sequential Mediation represented the theoretical core of this investigation, testing whether human-AI interaction experience influenced deep learning through the consecutive mediating roles of cognitive load and learning motivation. Bootstrap analysis confirmed significant sequential mediation [estimate = 0.118, SE = 0.020, 95% CI (0.085, 0.162)]. This positive indirect effect demonstrated that enhanced interaction experience promoted deep learning by first reducing cognitive load, which subsequently strengthened learning motivation, ultimately facilitating deep learning engagement.

Decomposition of total effects revealed that the sequential indirect pathway accounted for a meaningful proportion of the total relationship between human-AI interaction experience and deep learning. The variance accounted for (VAF) calculation indicated that indirect effects transmitted approximately 53% of the total effect, suggesting partial mediation wherein both direct and indirect pathways contributed to the overall relationship (Kio and Lau, 2017).

### 5.6 Final model presentation

**Figure 1:**
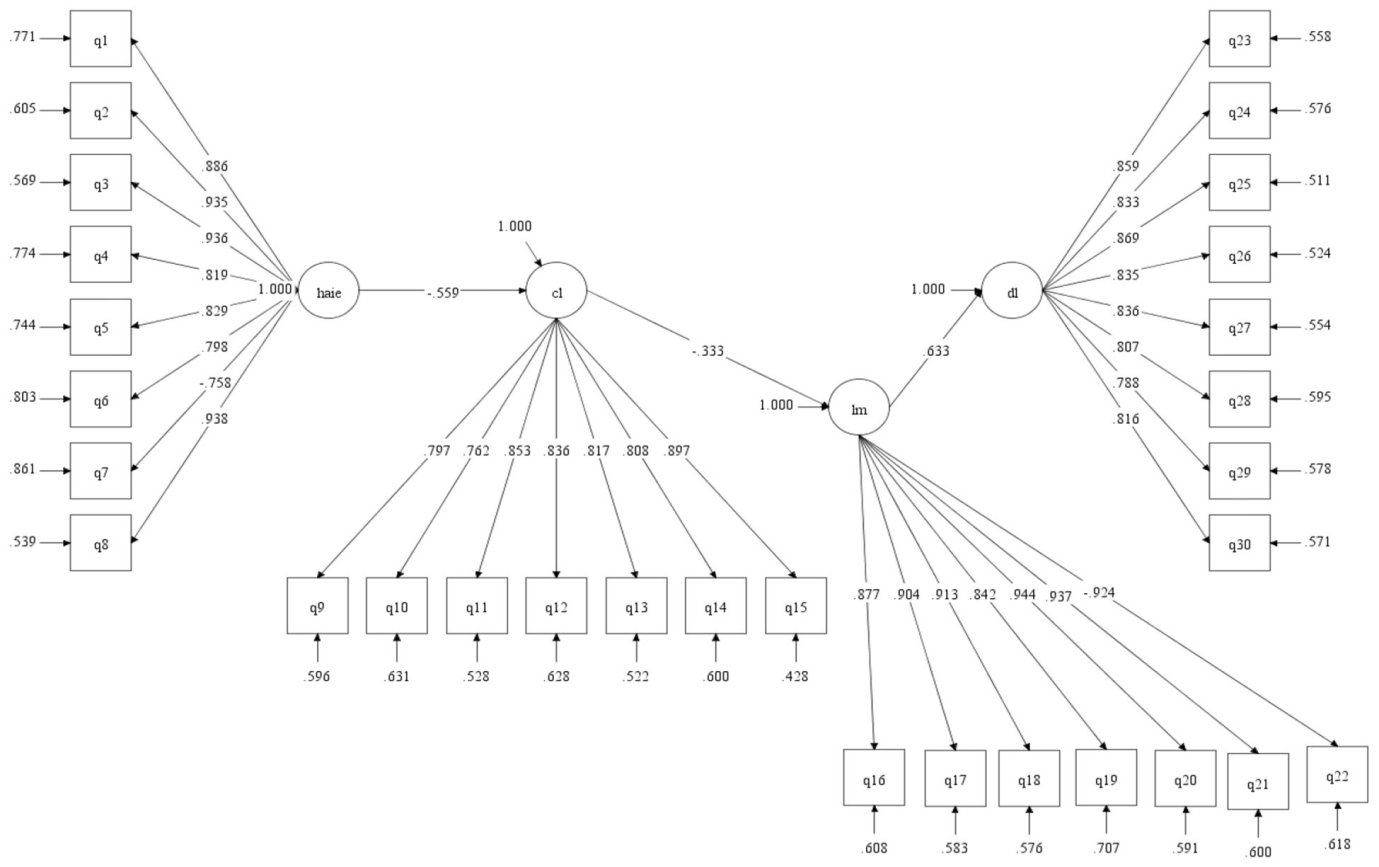
SEM path diagram.

Figure 1 presents the complete structural equation model with standardized path coefficients, significance levels, and explained variance proportions. The model illustrates the theoretically integrated pathway whereby human-AI interaction experience influences deep learning through sequential mediation. Superior interaction experience substantially reduces cognitive load (β = −0.488, *p* < 0.001), which in turn enhances learning motivation (β = −0.357, *p* < 0.001), ultimately promoting deep learning approaches (β =0.561, *p* < 0.001). The model achieved excellent fit indices and explained substantial variance in all endogenous variables, confirming the theoretical framework's empirical validity and explanatory power.

## 6 Discussion

### 6.1 Principal findings

Before interpreting the findings, it is important to acknowledge the correlational nature of this study. Given the cross-sectional design, the relationships identified reflect associations rather than causal effects. Terms such as ‘mediation' and ‘pathway' are used to describe statistical patterns consistent with theoretical predictions, but do not permit directional or causal inferences. The following discussion interprets results within these methodological constraints.

This investigation systematically examined the sequential mediation pathway through which human-AI interaction experience influences deep learning via cognitive load and learning motivation in AI-enhanced educational environments. The empirical findings provide comprehensive support for the proposed theoretical framework, addressing the three research questions and validating all hypothesized relationships.

Regarding RQ1, the study confirms that human-AI interaction experience is significantly associated with teachers' deep learning, operating through both direct and indirect pathways. The total effect magnitude indicates that interaction experience quality constitutes a meaningful determinant of learning approach adoption, though the relationship is predominantly mediated rather than direct. This finding extends existing research emphasizing technology acceptance and usability by demonstrating consequential effects on fundamental learning processes (Tawfik et al., 2024a).

RQ2 is addressed through validation of cognitive load and learning motivation as critical mediating mechanisms. Cognitive load emerged as a proximal consequence of interaction design quality (β = −0.488, *p* < 0.001), representing the most substantial path coefficient in the model. This robust effect magnitude corroborates Cognitive Load Theory's proposition that interface characteristics fundamentally influence extraneous load (Skulmowski and Xu, 2022). Subsequently, cognitive load demonstrated significant negative influence on learning motivation (β = −0.357, *p* < 0.001), confirming the resource depletion hypothesis whereby excessive cognitive demands undermine psychological resources necessary for autonomous engagement (Kalyuga and Liu, 2015). Learning motivation, in turn, functioned as a powerful predictor of deep learning approaches (β = 0.561, *p* < 0.001), consistent with Self-Determination Theory's emphasis on intrinsic motivation as a catalyst for deep cognitive processing (Pan, 2023).

RQ3 receives definitive empirical support through bootstrap analysis confirming significant sequential mediation [indirect effect = 0.118, 95% CI (0.085, 0.162)]. This result validates the theoretically integrated pathway: superior interaction experience reduces cognitive load, which preserves psychological resources for autonomous motivation, subsequently facilitating deep learning engagement. The sequential mediation accounts for 53% of the total effect variance, indicating partial mediation wherein both direct environmental factors and psychological mechanisms contribute to learning outcomes. This finding represents a theoretical advancement beyond previous research examining isolated relationships, demonstrating how technological, cognitive, and motivational factors operate synergistically within a unified explanatory framework.

The explained variance proportions provide additional insights into model effectiveness. Human-AI interaction experience accounted for 23.8% of cognitive load variance, suggesting that interface design constitutes a primary but not exclusive determinant of cognitive burden—task complexity and prior knowledge also contribute. The model explained 12.7% of motivation variance, indicating that while cognitive load influences motivation, additional factors merit investigation. Most notably, the framework explained 31.5% of deep learning variance, demonstrating substantial predictive utility while acknowledging that deep learning emerges from multiple determinants beyond the modeled constructs.

### 6.2 Theoretical contributions

This research advances educational technology scholarship through several theoretical contributions. First, the study extends Cognitive Load Theory by empirically establishing human-AI interaction experience as a consequential source of extraneous cognitive load in contemporary educational technologies (Schulz et al., 2025). While CLT traditionally focuses on instructional content and format, these findings demonstrate that interface design characteristics—usability, responsiveness, navigational clarity—impose cognitive demands that fundamentally influence learning processes. This extension is particularly significant given AI systems' unique characteristics: algorithmic opacity, adaptive feedback mechanisms, and personalized recommendation functions create distinctive cognitive challenges absent in conventional digital platforms (Jose et al., 2025). The substantial path coefficient (β = −0.488) underscores that interaction design optimization represents a primary leverage point for cognitive load management, warranting equivalent attention to content structuring in instructional design frameworks.

Second, the research deepens Self-Determination Theory by identifying cognitive resource availability as an antecedent condition for motivational processes (Akram and Li, 2024). Traditional SDT applications in educational contexts emphasize environmental supports for autonomy, competence, and relatedness needs. These findings reveal that cognitive load depletion represents a fundamental constraint on need satisfaction: when working memory capacity is overwhelmed by interface demands, learners experience diminished competence perceptions and reduced autonomous engagement regardless of environmental autonomy support. This cognitive-motivational linkage suggests that SDT must incorporate information processing constraints as boundary conditions for motivational dynamics, particularly in technology-mediated contexts where interface complexity varies substantially.

Third, the validated sequential mediation model integrates previously disparate theoretical frameworks—user experience design, Cognitive Load Theory, and Self-Determination Theory—within a unified explanatory structure (Salas et al., 2019). This theoretical synthesis demonstrates that effective AI educational systems require simultaneous optimization across technological (interface design), cognitive (load management), and motivational (need satisfaction) dimensions. The sequential pathway elucidates how these dimensions interconnect: technological quality influences psychological processes, which subsequently shape behavioral outcomes. This integration advances beyond additive multi-factor models toward process-oriented frameworks specifying causal mechanisms and temporal sequences.

Fourth, the findings contribute to learning experience design scholarship by empirically validating principles for optimizing educational technology effectiveness (Tawfik et al., 2024a). The results confirm that LXD must transcend aesthetic considerations to address fundamental cognitive and motivational processes. Specifically, usability and interaction quality function not merely as user satisfaction determinants but as critical mediators influencing learning depth through psychological mechanisms. This elevation of UX principles from peripheral concerns to central theoretical constructs justifies substantial investment in human-centered design methodologies within educational technology development.

Finally, the research enriches understanding of deep learning determinants by demonstrating that learning approaches emerge from complex interactions among environmental design, cognitive processing, and motivational states rather than direct instructional interventions alone (Grozev et al., 2024). The finding that motivation explains 31.5% of deep learning variance, substantially exceeding cognitive load's direct influence, underscores that fostering deep approaches requires sustained psychological engagement rather than mere cognitive capacity availability. This insight challenges instructional frameworks emphasizing knowledge transmission over motivational cultivation.

### 6.3 Practical implications

The empirical findings yield actionable guidance across multiple stakeholder domains. For educational technology designers, corresponds withdicate that interface optimization constitutes a primary leverage point for enhancing learning effectiveness. Specifically, designers should prioritize reducing extraneous cognitive load through simplified navigation structures, consistent interaction patterns, and minimized visual complexity (Syamala et al., 2025). AI system interfaces should provide transparent algorithmic explanations, predictable feedback mechanisms, and intuitive control options to reduce cognitive uncertainty. Adaptive personalization algorithms should balance sophistication with comprehensibility, avoiding algorithmic opacity that generates user confusion. Usability testing protocols should incorporate cognitive load assessment—not merely task completion metrics—to identify interface elements imposing unnecessary processing demands.

Cognitive load management strategies extend beyond interface design to encompass content presentation and activity structuring. Information should be chunked into manageable segments aligned with working memory capacity constraints (Meng et al., 2016). AI systems should provide cognitive offloading tools—note-taking functions, progress trackers, concept organizers—enabling learners to externalize information and reduce working memory burden. Learning pathways should sequence complexity progressively, ensuring foundational schema construction before introducing advanced concepts. Intelligent recommendation systems should maintain transparency in suggestion rationales, reducing cognitive load associated with evaluating algorithmic outputs.

Motivational support mechanisms require systematic integration within AI educational platforms. Systems should provide learner autonomy through customizable learning paths, pace control, and content selection options (Tan et al., 2024). Competence support necessitates adaptive difficulty adjustment preventing excessive challenge that overwhelms capacity or insufficient challenge that induces boredom. Feedback should emphasize mastery progression rather than normative comparison, fostering intrinsic motivation through competence validation. Social learning features—peer collaboration tools, discussion forums, collaborative problem-solving—may address relatedness needs, though empirical investigation of social functionality impacts remains necessary.

Deep learning facilitation requires explicit instructional scaffolding promoting critical thinking, knowledge integration, and metacognitive reflection. AI systems should incorporate prompts encouraging learners to question assumptions, identify conceptual relationships, and evaluate information critically rather than accepting algorithmic outputs passively (Wang and McWatt, 2023). Reflection tools should guide metacognitive awareness regarding learning strategies and comprehension monitoring. Authentic tasks requiring knowledge application in novel contexts should supplement content acquisition activities, promoting transfer capability characteristic of deep learning.

For instructional practitioners, findings suggest that effective AI integration requires attention to psychological mediation processes beyond technological implementation. Educators should receive professional development addressing cognitive load recognition and management strategies, enabling identification of teachers experiencing cognitive overwhelm. Training should emphasize motivational support techniques aligned with SDT principles—autonomy-supportive language, competence scaffolding, authentic learning contexts. Faculty should develop AI literacy enabling critical evaluation of educational technologies' cognitive and motivational affordances, facilitating informed selection and implementation decisions.

Institutional policymakers should recognize that AI educational technology effectiveness depends upon comprehensive ecosystem development rather than mere infrastructure acquisition. Investment in user experience research, iterative design testing, and continuous improvement mechanisms ensures technologies evolve based on empirical learning effectiveness data rather than vendor claims. Professional development resources should support faculty capacity for psychologically informed technology integration. Institutional learning analytics should monitor not only usage metrics but cognitive load indicators and motivational engagement patterns, enabling data-driven optimization. Ethical review processes should consider cognitive justice—ensuring AI systems do not systematically disadvantage learners with lower digital literacy or processing capacity through poor interface design.

### 6.4 Limitations and future directions

This study relies exclusively on self-reported measures, which capture perceived rather than observed learning processes. Deep learning, cognitive load, and motivation are complex constructs that may benefit from triangulation with behavioral indicators, learning analytics, or classroom observation data. The SEM approach, while statistically robust, provides a snapshot of relationships at a single time point and cannot capture the dynamic, evolving nature of human-AI interaction in educational practice. Recent methodological critiques have questioned the explanatory scope of survey-based SEM in technology adoption research, particularly in rapidly changing domains like AI. Future research should consider longitudinal designs, mixed-methods approaches, and integration of objective usage data to complement self-reported perceptions. Additionally, the inclusion of demographic and contextual control variables would strengthen internal validity and enable exploration of moderating effects.

Several methodological limitations warrant acknowledgment and suggest future research directions. The cross-sectional design precludes definitive causal inference despite theoretical and statistical support for proposed directional relationships (Homyamyen et al., 2025). Longitudinal investigations tracking interaction experience, cognitive load, motivation, and learning approaches across multiple time points would strengthen causal claims and illuminate temporal dynamics. Experimental designs manipulating interface characteristics while monitoring cognitive and motivational responses would provide rigorous causal evidence complementing correlational findings.

Self-report measurement introduces potential bias, particularly for cognitive load assessment where metacognitive accuracy varies across individuals (Li and Jiang, 2025). Future research should integrate objective cognitive load indicators—eye-tracking metrics, neurophysiological measures, secondary task performance—validating self-report instruments and enabling more precise measurement. Learning analytics data capturing behavioral engagement patterns—time-on-task, help-seeking frequency, navigation sequences—could supplement self-reported motivation. Deep learning assessment should incorporate performance-based evaluation requiring demonstration of critical thinking and knowledge transfer rather than relying exclusively on self-reported tendencies.

The single cultural context (higher educationteachers in one country) limits generalizability across educational levels and cultural contexts (Dong, 2025). Cross-cultural comparative research would determine whether cognitive-motivational pathways operate equivalently across cultural frameworks emphasizing different educational values. Extension to K-12 populations would assess whether developmental factors moderate the sequential mediation pathway. Professional education and workplace learning contexts warrant investigation to determine model transferability beyond higher education.

Unexamined moderators and mediators represent theoretical extension opportunities. Individual difference variables—prior technology experience, learning self-efficacy, cognitive styles—may moderate pathway strengths (Purnama et al., 2021). Instructional design characteristics—task authenticity, feedback specificity, social interaction opportunities—could function as additional mediators or moderators. AI system type heterogeneity (intelligent tutoring systems vs. adaptive content platforms vs. conversational agents) may produce differential effects on cognitive-motivational pathways, suggesting need for technology-specific investigations.

Temporal dynamics require longitudinal examination. Initial AI system exposure may generate elevated cognitive load that diminishes with familiarization, suggesting time-varying relationships. Motivational trajectories may exhibit honeymoon effects wherein initial enthusiasm wanes without sustained need support. Deep learning development likely unfolds gradually, requiring extended observation periods capturing authentic learning progressions rather than cross-sectional snapshots.

Future research should investigate intervention effectiveness based on theoretical model insights. Experimental studies comparing standard AI systems against cognitively optimized interfaces would test whether load reduction produces hypothesized motivational and learning benefits. Motivational scaffolding interventions could determine whether need-supportive features enhance outcomes when controlling for cognitive load. Implementation science approaches would examine barriers and facilitators to translating empirical findings into authentic educational practice.

Theoretical extensions might integrate additional frameworks. Social Cognitive Theory constructs—observational learning, self-efficacy, outcome expectations—could complement SDT in explaining motivational processes (An et al., 2022). Expectancy-Value Theory would illuminate how interaction experience influences task value perceptions and success expectations. Embodied cognition perspectives might enrich understanding of how interface modalities (visual, auditory, haptic) differentially influence cognitive load and engagement. These theoretical integrations would advance toward comprehensive models of technology-enhanced learning encompassing cognitive, affective, social, and contextual dimensions.

## 7 Conclusion

This investigation empirically validated a sequential mediation model wherein human-AI interaction experience influences deep learning through cognitive load and learning motivation in AI-enhanced educational environments. Employing structural equation modeling with data from 570 higher education teachers, the study confirmed all hypothesized relationships: superior interaction experience substantially reduced cognitive load (β = −0.488, *p* < 0.001), which enhanced learning motivation (β = −0.357, *p* < 0.001), subsequently promoting deep learning approaches (β = 0.561, *p* < 0.001). Bootstrap analysis established significant sequential mediation [indirect effect = 0.118, 95% CI (0.085, 0.162)], demonstrating that technological design quality influences learning depth through interconnected psychological mechanisms rather than direct effects alone.

The theoretical contribution lies in integrating Cognitive Load Theory, Self-Determination Theory, and user experience research within a unified explanatory framework, establishing that effective AI educational systems require simultaneous optimization across technological, cognitive, and motivational dimensions. Practically, findings provide evidence-based guidance for interface design prioritizing cognitive load reduction, motivational scaffolding supporting psychological need satisfaction, and instructional strategies fostering deep learning engagement. As educational institutions navigate digital transformation, this research underscores that technology effectiveness depends fundamentally upon human-centered design attending to learners' cognitive processing constraints and motivational prerequisites for meaningful knowledge construction.

## Data Availability

The raw data supporting the conclusions of this article will be made available by the authors, without undue reservation.
